# In Vivo and In Vitro Antidiabetic Efficacy of Aqueous and Methanolic Extracts of Orthosiphon Stamineus Benth

**DOI:** 10.3390/pharmaceutics15030945

**Published:** 2023-03-14

**Authors:** Najlaa Bassalat, Sleman Kadan, Sarit Melamed, Tamar Yaron, Zipora Tietel, Dina Karam, Asmaa Kmail, Mahmud Masalha, Hilal Zaid

**Affiliations:** 1 Faculty of Sciences, Arab American University, Jenin P.O. Box 240, Palestine; 2 Faculty of Medicine, Arab American University, Jenin P.O. Box 240, Palestine; 3 Qasemi Research Center, Al-Qasemi Academic College, P.O. Box 124, Baqa El-Gharbia 3010000, Israel; 4 Department of Food Science, Gilat Research Center, Agricultural Research Organization, M.P. Negev, Gilat 8531100, Israel; 5 Faculty of Science, Beit Berl College, Kfar Saba 4490500, Israel

**Keywords:** *Orthosiphon stamineus*, GLUT4, anti-oxidant, diabetes mellitus, phytochemicals

## Abstract

*Orthosiphon stamineus* is a popular folk herb used to treat diabetes and some other disorders. Previous studies have shown that *O. stamineus* extracts were able to balance blood glucose levels in diabetic rat animal models. However, the antidiabetic mechanism of *O. stamineus* is not fully known. This study was carried out to test the chemical composition, cytotoxicity, and antidiabetic activity of *O. stamineus* (aerial) methanol and water extracts. GC/MS phytochemical analysis of *O. stamineus* methanol and water extracts revealed 52 and 41 compounds, respectively. Ten active compounds are strong antidiabetic candidates. Oral treatment of diabetic mice with *O. stamineus* extracts for 3 weeks resulted significant reductions in blood glucose levels from 359 ± 7 mg/dL in diabetic non-treated mice to 164 ± 2 mg/dL and 174 ± 3 mg/dL in water- and methanol-based-extract-treated mice, respectively. The efficacy of *O. stamineus* extracts in augmenting glucose transporter-4 (GLUT4) translocation to the plasma membrane (PM) was tested in a rat muscle cell line stably expressing myc-tagged GLUT4 (L6-GLUT4myc) using enzyme-linked immunosorbent assay. The methanol extract was more efficient in enhancing GLUT4 translocation to the PM. It increased GLUT4 translocation at 250 µg/mL to 279 ± 15% and 351 ± 20% in the absence and presence of insulin, respectively. The same concentration of water extract enhanced GLUT4 translocation to 142 ± 2.5% and 165 ± 5% in the absence and presence of insulin, respectively. The methanol and water extracts were safe up to 250 µg/mL as measured with a Methylthiazol Tetrazolium (MTT) cytotoxic assay. The extracts exhibited antioxidant activity as measured by 2,2-diphenyl-1-picrylhydrazyl (DPPH) assay. *O. stamineus* methanol extract reached the maximal inhibition of 77 ± 10% at 500 µg/mL, and *O. stamineus* water extract led to 59 ± 3% inhibition at the same concentration. These findings indicate that *O. stamineus* possesses antidiabetic activity in part by scavenging the oxidants and enhancing GLUT4 translocation to the PM in skeletal muscle.

## 1. Introduction

Type 2 diabetes (T2DM) is considered a global health issue threatening the life of 537 million people worldwide in 2021. Diabetic cases are estimated to rise up to 784 million by 2045 [1]. T2DM is responsible for a 5% increase in premature mortality due to its complications, which start with hyperglycemia and proceed to a combination of resistance to insulin action, insufficient insulin secretion, and excessive glucagon breakdown and secretion [2]. Diabetes is also considered a major cause of blindness, kidney failure, heart attacks, stroke, and lower limb amputation. T2DM is a multifactorial disorder that can be triggered by aging and genetic factors, environmental factors, and factors related to the patient’s lifestyle, such as diet, physical activity, and obesity [3].

Increased glucose levels in bloodstream stimulate secretion of insulin from the islets of Langerhans in the pancreas. Insulin is the responsible hormone for regulation of circulation glucose levels by increasing glucose transport into adipose and muscle tissues and by suppressing the hepatic glucose production. Binding of the insulin to its receptors on cell surfaces induces the translocation of glucose transporter-4 (GLUT4) from intracellular vessels to plasma membranes, which results in diffusion of glucose in muscle, hepatocytes and adipocytes [4]. Insulin binds to the β-subunit of the insulin receptor (IR), leading to autophosphorylation and then recruitment of the insulin receptor substrate-1 (IRS-1), which in turn activates phosphatidylinositol 3-kinase (PI3K). PI3-K phosphorylates phosphatidylinositol-4, 5-bisphosphate (PIP2) to yield phosphatidylinositol-3, 4, 5-triphosphate (PIP3). PIP3 activates Akt (protein kinase B), which then phosphorylates many substrates, including Akt substrate of 160 kDa (AS160), leading to its inhibition and thus augmenting GLUT4-containing vesicle translocation and fusion with the plasma membrane (PM) [5]. GLUT4 translocation to the PM is also triggered by AMP-activated protein kinase (AMPK) under certain conditions such as muscle contraction, increased cellular [AMP]/[ATP] ratio and deprivation of glucose or oxygen. AMPK activation in skeletal muscle promotes GLUT4 trafficking to the PM and enhanced glucose uptake in the insulin independent pathway [4,5].

Medicinal plants are traditionally used in folk medicine as natural healing remedies with therapeutic effects for many diseases, including diabetes. Antidiabetic herbs balance blood glucose and delay the progression of diabetic complications. More than 800 plant species worldwide are reported as potentially antidiabetic herbs [6].

*Orthosiphon stamineus* Benth. (Lamiaceae) is a perennial herb found in tropical and subtropical regions [7]. *O. stamineus* is used in folk medicine as an antidiabetic and diuretic and for treating abdominal pain, kidney and bladder inflammation, edema, and gout [8]. Pharmacological studies have shown antimicrobial, antioxidant, hepatoprotective, antigenotoxic, antiplasmodial, cytotoxic, cardioactive, anti-inflammatory and antidiabetic activities [8,9]. Reports have shown that *O.* *stamineus* contains a variety of groups of phytochemicals such as flavones and other polyphenols, bioactive proteins, glycosides, volatile oils, as well as large quantities of potassium [9].

One animal study has shown that the water extract of *O. stamineus* has hypoglycemic effects on diabetic rats [10]. In a recent study, 80% ethanol extract (about 0.4 g/kg) reduced blood glucose levels in an oral glucose tolerance test in normal C57BL/6J mice and high-fat-diet (HFD) C57BL/6 mice after 1.5 [11] and 8 weeks [12] administration of the extract, respectively. Water extract of *O. stamineus* was also administrated orally (0.5 g/kg) to normal and diabetic rats loaded with glucose. In normal rats, the aqueous extract reduced plasma glucose after 1 h of glucose loading by 15% and 21% in diabetic rats, respectively [13].

Yet the antidiabetic mechanism of action of these plant extracts is not known. According to a recent systematic review [7], no study was published on the effect of *O. stamineus* extracts on GLUT4 translocation and activity. This study thus aims to examine the effect of *O. stamineus* extracts on GLUT4 translocation to the PM in L6 skeletal muscle cell line, its antioxidant scavenging activity, and its chemical composition

## 2. Materials and Methods

### 2.1. Materials

𝛼-MEM (modified Eagle’s medium), fetal bovine serum, and all other tissue culture reagents were purchased from biological industries (Beit Haemek, Israel). Horseradish-peroxidase (HRP-) -conjugated goat anti-rabbit antibodies were obtained from Promega (Madison, WI, USA). Polyclonal anti-myc (A-14) and other standard chemicals were purchased from Sigma-Aldrich (Saint Louis, MO, USA).

### 2.2. Plant Extract Preparation

The aerial parts of *Orthosiphon stamineus* Benth were purchased from a medicinal plants trader in Nablus, Palestine. The plant was identified by Prof. Nidal Jaradat (An-Najah National University, Nablus, Palestine). Two extracts were prepared: in water and in methanol. Forty grams of air-dried aerial parts of *O. Stamineus* was powdered and packed in an Erlenmeyer flask with 200 mL solvent. The flasks were then sonicated for 2 h at 60 °C (Elmasonic, Singen, Germany) and left in dark glass bottles for 24 h for complete extraction. The yield of the extract in methanol and water was 10.5% and 12.7%, respectively. The stock extracts were kept at −20 °C in air-tight glass containers.

### 2.3. Silylation Derivatization

One mL of the water and methanol extract was transferred to a glass vial, and the solvents were evaporated under a gentle stream of nitrogen at ambient temperature, and 150 µL of *N,O*-Bis (trimethylsilyl) trifluoroacetamide (BSTFA) containing 1% trimethylchlorosilane reagent used for GC silylation derivatization (>99%, Sigma-Aldrich) was added to each dry *O. Stamineus* crude extract followed by heating up to 70 °C for 20 min [14].

### 2.4. Gas Chromatography—Mass Spectrometry Analysis and Compounds Identification

One μL of each silylated sample was injected into the gas chromatograph (GC) coupled with mass spectrometer detector (MS) as previously described by our group [15]. Component relative percentages of the samples were calculated from the GC peak areas. NIST GC/MS Library and mass spectra from the literature were used to annotate the compounds.

### 2.5. Cell Growth and Treatment

Cells from the rat L6 muscle cell line stably expressing myc-tagged GLUT4 (L6-GLUT4myc) were purchased from (Kerafast, Boston, MA, USA). The cells were grown at 37 °C, 95% air, and 5% CO_2_ in a-MEM supplemented with 10% fetal calf serum (FCS), 100 U/mL penicillin, and 100 µg/mL streptomycin.

### 2.6. MTT Cytotoxic Assay

The Methylthiazol Tetrazolium (MTT) assay was used to detect the viability of the cells as described in [16]. This assay relies on the colorimetric change from yellow to purple, indicating that the cells are active. On the first day, cells with a density of 2 × 10^4^ were seeded in 96 well-plates, each well containing 100 µL of medium. The cells were incubated for 24 h in an incubator (37 °C and 5% CO_2_). On the next day, 100 µL of the *O. Stamineus* extracts (water and methanol) were added to each well at increasing concentrations up to 1 mg/mL and incubated for 20 h. The old medium was removed and a fresh medium containing 0.5 mg/mL MTT was added into each well and incubated for 4 h. Medium containing MTT was removed and 100 µL of isopropanol/HCl (1 mM HCl in 100% isopropanol) were added to each well. Absorbance at 570 nm was measured using a plate reader. Experiments were repeated three times, with six replicates each time.

The following formula was used to detect the viability of the cells:

Percent viability = (A570 nm of plant extract treated cells/A570 of untreated cells) × 100%

### 2.7. GLUT4 Translocation

Surface myc-tagged GLUT4 was measured in intact cells as described previously [14]. Briefly, L6-GLUT4 myc cells were seeded in 24 well-plates and incubated for 24 h. *O. Stamineus* extracts (water and methanol) were added to the cells for 20 h and followed by serum starvation for 3 h and treated with or without 1 µM insulin for 20 min. The cells were washed twice with ice-cold PBS, fixed with 3% paraformaldehyde for 15 min, then blocked with 3% (*v*/*v*) goat serum for 10 min, incubated with polyclonal anti-myc antibody (1:200) for 1 h at 4 °C, washed ten times with PBS and incubated with goat-anti-rabbit secondary antibody conjugated with horseradish peroxidase (1:1000) for 1 h at 4 °C, then washed ten times with PBS at room temperature. One milliliter of o-phenylenediamine dihydrochloride reagent was added to each well and incubated in the dark at room temperature for 20–30 min and the reaction was stopped by adding 0.5 mL of 3 M HCl. The absorbance was measured by using a spectrophotometer at 492 nm. Background absorbance obtained from 3 wells in each 24-well plate untreated with anti-myc antibody was subtracted from all values.

### 2.8. DPPH Scavenging Activity

2,2-diphenyl-1-picrylhydrazyl (DPPH) free radical scavenging ability of the extracts was tested as described by Blois [17] with slight modifications. The hydrogen atom donating ability of the plant extractives was determined by the decolorization of methanol solution of DPPH which produces violet/purple color in methanol solution and fades to yellow in the presence of antioxidants.

DPPH (0.002% *w*/*v*) dissolved in methanol was mixed with *O. stamineus* extract or Trolox as a positive control at a 1:1:1 ratio. A negative control solution was prepared by mixing the mentioned DPPH solution with methanol in a 1:1 ratio. The mixtures were incubated at room temperature in the darkness for 30 min. The color density was determined by a Spectrophotometer at 517 nm (Jenway 72000, Cole-Palmer, Vernon Hills, IL, USA). The antioxidant activity of Trolox and *O. stamineus* extract was calculated using the following formula:

% Inhibition of DPPH activity = (Blank absorbance − extract absorbance/Blank absorbance) × 100%

### 2.9. Animals and Induction of Type-2 Diabetes

The experimental protocols, ethical procedures, and policies were authorized according to the Animal Care and Use Committee of Arab American university. In the current study, male C57BL/6 mice (14 weeks of age and weighing approximately 30 g) obtained from the Arab American university experimental animals care facility were used. The animal facility maintained an environment temperature of 25 ± 2 °C, a humidity of 55 ± 5%, with a 12 h controlled light/dark cycle. Free access to tap water and standard laboratory food was given to the mice and separated to groups 1 week before starting the experimental part.

**Induction of diabetes:** Mice were treated with streptozotocin (STZ) (40 mg/kg) in 50 mM sodium citrate buffer (pH, 4.5) for 5 consecutive days via the intraperitoneal route. To prevent fatal hypoglycemia, mice freely received 10% sucrose water for 5 days after STZ treatment. On experimental day 14 (9 days after the last STZ injection), the blood sugar levels were measured with a diabetes test strips in a glucometer apparatus (Abbott, Abbott Park, IL, USA). Mice with a blood glucose levels more than 200 mg/dL were considered diabetic and taken for further experimentation procedures [18].

**Grouping of animals:** All the experimental mice were given an essential diet during the experimental period and separated into six groups (n = 4): Control non-diabetic groups without and with WOS and MOS and STZ-induced diabetic mice without and with WOS and MOS. The extracts were diluted in water daily and administered by gavage at a concentration of 100 mg/kg.

### 2.10. Statistical Analysis

The data were normally distributed and variables were expressed as mean ± SEM. T test was used to investigate statistically significant differences. A level of *p* < 0.05 was accepted as statistically significant. Statistical analyses were conducted using SPSS version 23.0 for Windows.

## 3. Results and Discussion

This study focused on testing the chemical composition, cytotoxicity, and antidiabetic activity of two *O. stamineus* extracts: water (WOS) and methanol (MOS).

### 3.1. Toxicity of O. stamineus Extracts

**Figure 1:**
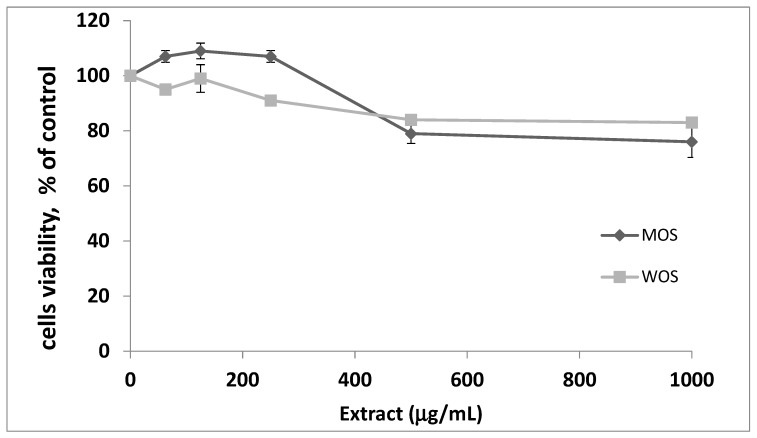
Effect of *O. stamineus* methanol (MOS) and water (WOS) extract on cell viability examined by MTT assay. L6-GLUT4myc cells (20,000 cell/well) were seeded in 96-well plate and were exposed to the extract for 24 h. Values given represent means ± SEM (% of untreated control cells) of three independent experiments carried out in triplicates.

MTT assay was performed to define the non-toxic concentrations of the two *O. stamineus* extracts: water (WOS) and methanol (MOS). Extract concentrations that led to more than 90% of cell viability were considered safe, non-toxic concentrations. It was shown that concentrations up to 250 µg/mL were safe for both WOS and MOS extracts (Figure 1). Therefore, the efficacy experiments of WOS and MOS were performed up to 250 µg/mL.

### 3.2. Effects of O. stamineus Extracts on GLUT4 Translocation

**Figure 2:**
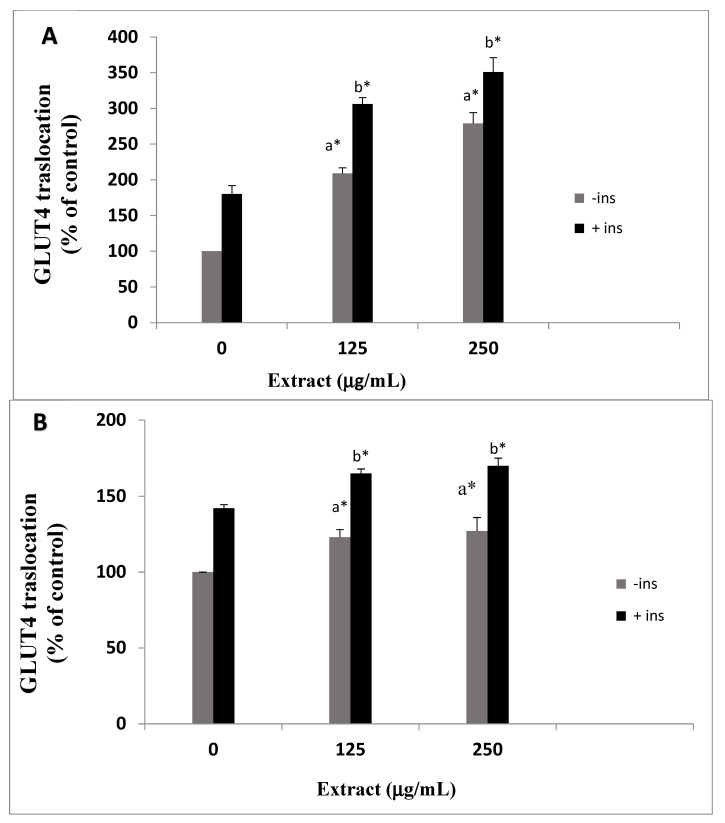
GLUT4 translocation to the plasma membrane. L6-GLUT4myc cells (150,000 cell/well) were seeded in a 24-well plate and were exposed to WOS (**A**) and MOS (**B**) without (−) or with (+) insulin as described in the methods. Surface *myc*-tagged GLUT4 density was quantified using the antibody coupled colorimetric assay. Values given represent means ± SEM (relative to untreated control cells) of three independent experiments carried out in triplicates. Statistical significance: (a) compared with (-ins) control group, (b) compared with (+ins) control group.

In type 2 diabetes, GLUT4 translocation to the plasma membrane is impaired. Some antidiabetic medicinal plants can increase the translocation of GLUT4 [19]. Therefore, the effect of the two extracts (WOS and MOS) on the translocation of GLUT4 to the plasma membrane was measured by the GLUT4 translocation assay as described in the methods in the presence and absence of insulin. WOS extract enhanced GLUT4 translocation slightly; at 250 µg/mL, the translocation increased from 100% to 142 ± 2.5% and 165 ± 5% with and without insulin, respectively (Figure 2A). MOS extract exhibited a more potent effect on GLUT4 translocation. GLUT4 translocation reached 209 ± 8% and 306 ± 9% in cells exposed to 125 µg/mL MOS in the presence and absence of insulin, respectively. GLUT4 translocation increased more when exposed to 250 µg/mL and reached 279 ± 15% and 351 ± 20% in the absence and presence of insulin, respectively (Figure 2B).

The extent of increase in insulin-stimulated GLUT4 translocation, as depicted in Figure 2, was additive to that of basal GLUT4 translocation in *O. stamineus*-exposed cells, especially MOS. This result suggests a possible additive efficacy between *O. stamineus* active ingredients and insulin. Consecutively, *O. stamineus* active phytochemicals might enhance GLUT4 translocation in a non-insulin dependent pathway, such as the AMP-activated protein kinase (AMPK) pathway. It is possible that *O. stamineus* active compounds might possess “insulin-sensitizing” activity.

### 3.3. DPPH Scavenging Activity

**Figure 3:**
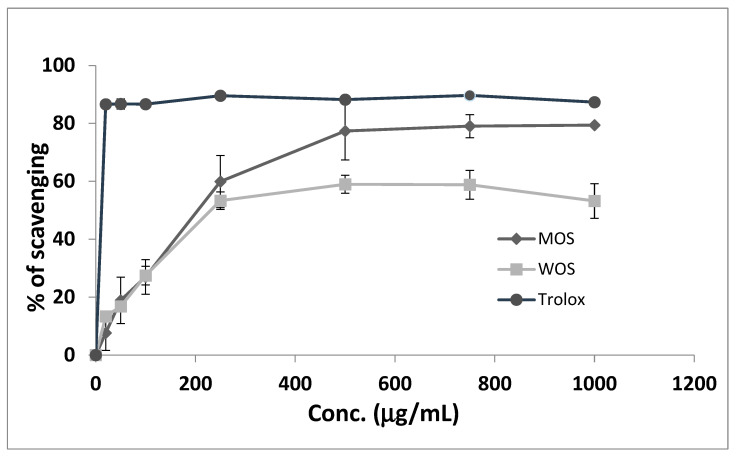
Determination of DPPH radical scavenging activity of MOS and WOS. Trolox was used as a positive control. All experiments were performed in triplicates. Data are expressed as mean ± SEM.

The effect of antioxidants on DPPH is believed to be due to their hydrogen-donating ability [17]. The ability of the WOS and MOS extracts to act as antioxidants was tested by the DPPH scavenging activity assay. WOS and MOS scavenging activity was tested up to 100 µg/mL and reached 53.3 ± 4.8% and 79 ± 0.6%, respectively (Figure 3). Trolox was used as a positive control and led to maximum scavenging activity at around 20 µg/mL. MOS reached the maximal inhibition of 77 ± 10% at 500 µg/mL, while WOS led to 59 ± 3% at the same concentration (Figure 3). None of the extracts led to maximal scavenging like Trolox, yet MOS was more efficient in DPPH scavenging, indicating its higher content of antioxidant compounds (Table 1).

### 3.4. Effect of O. stamineus Extracts on Diabetic Mice Blood Glucose Levels and Mass

**Figure 4:**
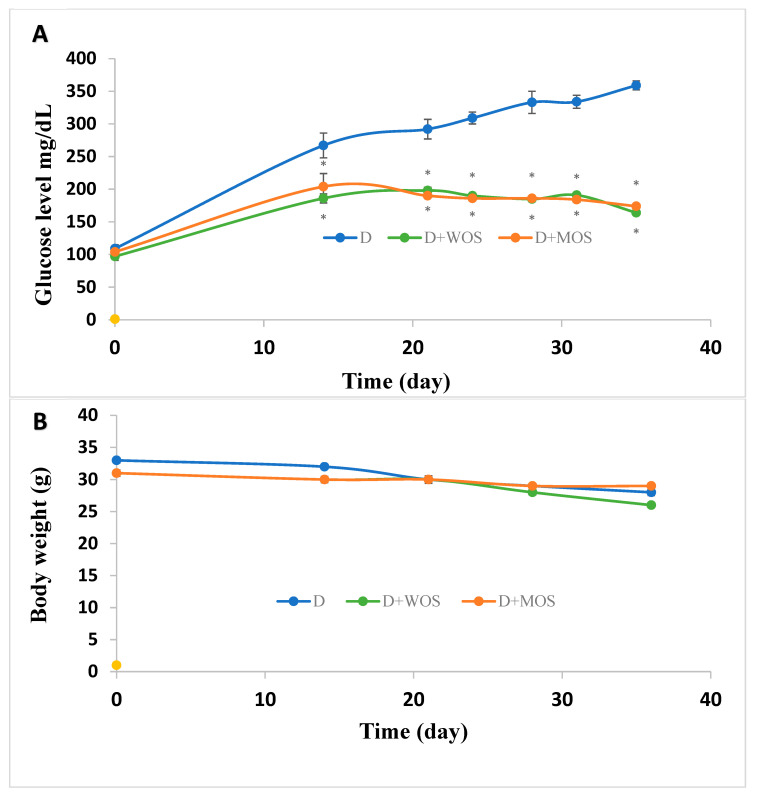
The effect of oral administration of 100 mg/kg WOS and MOS on blood glucose levels (**A**) and body weight (**B**) of STZ-induced diabetic mice. Values are expressed as mean ± SEM. * *p* < 0.05, significant as compared with diabetic group.

The effects of WOS and MOS on blood glucose levels in the STZ-injected mice are shown in Figure 4A. The treatment with *O. stamineus* extracts for 3 weeks resulted significant reductions in blood glucose levels compared to diabetic control group (*p* < 0.05). The effect was significantly appreciated after 1 week of administration. At day 36, the blood glucose level in the diabetic control group mice was 359 ± 7 mg/dL compared to 164 ± 2 mg/dL and 174 ± 3 mg/dL in WOS and MOS treated mice, respectively (Figure 4A). Blood glucose levels in non-diabetic mice were 104 to 109 mg/dL with and without WOS and MOS during the experiment period. Moreover, WOS and MOS did not affect the non-diabetic mice mass, which was 31 to 34 gr during the experiment period. As expected, diabetic mice mass was reduced from 32 ± 0.4 gr at the first day of the experiment to 28 ± 0.5 gr at day 36. MOS but not WOS rescued the mice mass as mice mass treated with MOS decreased only from 31 ± 0.5 gr to 29 ± 0.2 gr. WOS-treated mice mas decreased from 31 ± 0.3 to 26 ± 0.3 gr (Figure 4B).

STZ is widely used as a diabetogenic agent in mice [18]. It is a potent alkylating agent that enters the pancreatic β cells via GLUT2 and enhances DNA methylation and enhances hydrogen peroxide generation, causing DNA fragmentation and apoptosis and necrosis induction. STZ thus leads to insulin depletion leading to hyperglycemia. WOS and MOS significantly lowered blood glucose levels by 54% and 57%, respectively, compared with diabetic non-treated mice. MOS also maintained the diabetic mice mass. It decreased only by 6% at day 36 compared to 15% and 16% in the diabetic non-treated group and WOS-treated group, respectively. Those results are in line with the in vitro GLUT4 translocation results, where MOS was more effective in augmenting GLUT4 to the PM surface (Figure 2). GC/MS tests also showed that MOS is more rich in antidiabetic phytochemicals compared with WOS.

### 3.5. Chemical Analysis of O. stamineus Extracts

Phytochemical screening using GC/MS revealed 52 compounds in WOS (Table 1) and 41 in MOS (Table 2), including sterols, esters, phenolic compounds, saturated and unsaturated fatty acids, and aromatic compounds. Fourteen components, namely, phosphonic acid, glycerol, butanedioic acid, glyceric acid, pinitol, fructose, galactose, mannitol, gluconic acid, myo-inositol, glyceryl-glycoside, uridine, sucrose, and trehalose, were conjoined in the two extracts.

**Figure 5:**
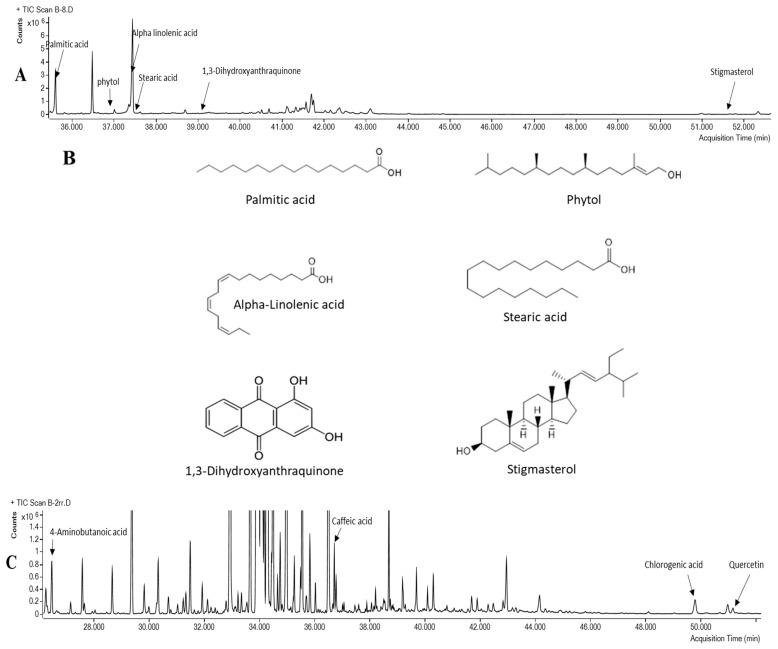

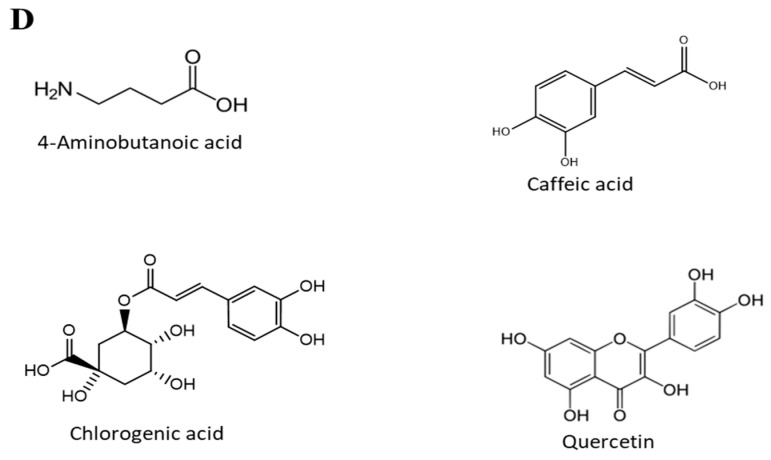
Total ion chromatogram (TIC) of *O. stamineus* methanol (**A**) and water (**C**) extract. Chemical structure of the identified antidiabetic components in *O. stamineus* methanol and water extract is depicted in (**B**) and (**D**), respectively.

The recognized antidiabetic compounds, especially those previously reported to enhance glucose uptake and increase GLUT4 activity, are highlighted in bold in Table 1 and Table 2, and their chemical structure is drawn in the GC/MS chromatogram (Figure 5). In MOS, palmitic acid, phytol, alpha-linolenic acid, stearic acid, 1,3-dihydroxyanthraquinone, and stigmasterol (Table 1 and Figure 5B) are reported to enhance glucose disposal. Palmitic acid augmented GLUT4 translocation in muscle cells [14], and phytol was reported to increase AS160 and GLUT4 gene expression and activate the PI3K/Akt signaling pathway in mouse white adipose tissue [20]. Alpha-linolenic acid lowered blood glucose levels in diabetic mice as it increased GLUT4 amount at the muscle membrane [21]. Stearic acid enhanced basal glucose uptake in myotubes [22]. 1,3 Dihydroxyanthraquinone enhanced glucose uptake in C2C12 muscle cells in an AMPK-signaling-dependent pathway [23]. Stigmasterol augmented GLUT4 translocation and expression in L6 muscle cells [24]. Among the detected compounds in MOS was erythritol. It is 60–70% as sweet as sucrose; however, it provides only 6% of the calories in an equal amount of sugar and does not affect blood sugar levels [25].

In WOS, four compounds were found to be antidiabetic, namely, caffeic acid, chlorogenic acid, 4-aminobutanoic acid, and quercetin (Table 2 and Figure 5D). Only the last two compounds were reported to enhance GLUT4 translocation. 4-aminobutanoic, also known as gamma-aminobutyric acid (GABA), improved insulin resistance in diabetic patients by increasing the expression of GLUT4 [26]. Quercetin increased expression of GLUT4 [5]. Caffeic acid and chlorogenic acid were reported to possess antidiabetic activity, yet their activity was not associated directly with GLUT4 activity. Recently, caffeic acid was shown to decrease blood glucose levels and improve glucose tolerance in diabetic rats in an unknown mechanism [27]. Chlorogenic acid reduced insulin resistance and modulated glucose uptake in HepG2 cell line [28].

To our best of knowledge, this is the first reported study that compares water and methanol-based extracts from *O. stamineus* arial part (leaves and barks) in terms of detected antidiabetic active ingredients, antioxidant activity, and antidiabetic activity in vitro and in vivo at low doses (100 mg/kg). Others have treated diabetic mice and rats with up to 1000 mg/kg. For instance, in STZ-induced diabetic rats, only the group treated with 1000 mg/kg of the 50% ethanolic extract of *O. stamineus* [29] and water extract at 500 mg/kg [13] showed significantly lower plasma glucose levels. Others reported 200 and 400 mg/kg of ethanol extract of *O. stamineus* reduced fasting blood glucose levels in high-fat-diet (HFD) C57BL/6 mice after 8-week administration of the extract [12]. In the present study, the WOS and MOS reduced the diabetic mice blood glucose levels at low doses, and its antidiabetic activity was appreciated the first week of treatment.

The present results showed that *O. stamineus* methanol extract (and, to a lesser extent, the water extract) significantly enhanced GLUT4 translocation to the PM at non-toxic concentrations, reduced blood sugar levels of diabetic mice, and possessed antioxidant activity. Thus, *O. stamineus* extract may be beneficial for diabetes treatment. Ten reported effective antidiabetic constituents were detected in the extracts, especially in the MOS extract, which is in line with our current findings that MOS is more effective in augmenting GLUT4 translocation. It is crucial to separate *O. stamineus* detected phytochemicals in order to identify their cellular/molecular target and point out their specific antidiabetic mechanism and cellular pathways.

## 4. Conclusions

*O. stamineus* water and especially methanol extracts significantly reduced the plasma glucose concentrations of STZ-induced diabetic mice. Concomitantly, methanol extract was more efficient in augmenting GLUT4 translocation to the PM of L6 myocytes. Among the compounds detected, 10 are reported to enhance either GLUT4 transport or translocation to the PM. Moreover, *O. stamineus* extracts exhibited antioxidant activity that might be associated with its antidiabetic activity and glucose disposal. *O. stamineus* might be considered an antidiabetic agent once its activity is tested and proven on diabetic subjects.

## Figures and Tables

**Table 1:** Phytochemicals of *O. stamineus* from methanol extract verified by GC/MS.

Peak	Name	R_t_	% Area	Match Factor
1	Propane-1,2-diol	12.46	0.081	67.8
2	Lactic Acid	13.42	0.043	90.5
3	1-Heptanol	13.60	0.008	66.9
4	Phosphonic acid	19.99	0.557	86.8
5	Glycerol	20.11	13.714	98.3
6	Butanedioic acid (Succinic acid)	20.93	0.384	96.9
7	Glyceric acid	21.59	0.407	97
8	Erythritol	26.11	0.034	93.3
9	Threitol	26.29	0.177	97.4
10	4-Hydroxybenzeneacetic acid	28.87	0.207	87.9
11	Neophytadiene	32.75	0.153	83.6
12	Pinitol	33.23	2.331	82.7
13	Saccharide -unknown	33.52	2.613	67.4
14	Fructose	34.07	29.749	95.7
15	Galactose	34.49	1.049	80
16	Mannitol	34.71	18.749	92.3
17	Sorbitol	34.97	11.994	95.9
18	saccharide -unknown	35.07	0.890	86.3
19	Gluconic acid	35.82	0.061	88.9
20	Palmitic Acid	35.61	2.584	98.4
21	Myo-Inositol	36.48	2.672	98.1
22	Phytol	37.01	0.173	90.5
23	9,12-Octadecadienoic acid (Linoleic acid)	37.36	0.632	73
24	alpha-Linolenic acid	37.44	6.102	98.4
25	Stearic acid	37.62	0.161	92.3
26	Glyceryl-glycoside	38.69	0.262	91.7
27	1,3-Dihydroxyanthraquinone	39.27	0.022	67.5
28	Uridine	39.66	0.064	68.9
29	Sucrose	41.75	0.812	82.1
30	Trehalose	43.10	0.536	77.5
31	1-Octacosanol	48.91	0.037	81
32	alpha-Tocopherol	49.16	0.010	77.1
33	Campesterol	51.80	0.065	80.8
34	Stigmasterol	52.33	0.338	91.6
35	beta-Sitosterol	53.22	0.547	97.7
36	Sterol-unknown	53.31	0.260	89.3
37	Sterol-unknown	53.41	0.182	65.8
38	Sterol-unknown	53.78	0.208	84.5
39	Sterol-unknown	53.89	0.275	88.1
40	Sterol-unknown	54.85	0.410	76.8
41	Sterol-unknown	54.97	0.448	89.5

**Table 2:** Phytochemicals of *O. stamineus* from water extract verified by GC/MS.

Peak	Name	R_t_	% Area	Match Factor
1	Glycin	12.24	0.02	87.7
2	Alanine	14.75	0.09	96
3	Leucine	16.23	0.03	86
4	Proline	16.70	0.39	91.4
5	Isoleucine	16.84	0.03	84.1
6	Malonic acid	17.87	0.07	95.3
7	Valine	18.21	0.12	96.2
8	Serine	19.34	0.07	86.4
9	Phosphonic acid	19.99	2.81	90.1
10	Glycerol	20.09	1.94	98.5
11	Butanedioic acid (Succinic acid)	20.91	0.28	98.3
12	Glyceric acid	21.64	4.05	97.5
13	2-Butenedioic acid (Fumaric acid)	21.79	0.04	95.4
14	Threonine	23.07	0.19	95.9
15	Aspartic acid	23.77	0.05	94.7
16	Malic acid	25.76	19.19	97.8
17	5-Oxoproline	26.25	0.19	96.8
18	4-Aminobutanoic acid (GABA)	26.46	0.35	97.1
19	Phenylalanine	26.64	0.02	79
20	Threonic acid	27.14	0.07	99.1
21	Erythronic acid	27.57	0.32	85.4
22	Glutaric acid	28.65	0.27	88
23	Tartaric acid	29.37	2.38	98.3
24	Asparagine	29.81	0.18	96.1
25	2-amino-Adipic acid	30.78	0.02	85.2
26	Ribose	31.48	0.02	82.1
27	Glutamine	31.92	0.17	96.1
28	Citric acid	32.94	7.54	87.1
29	Pinitol	33.22	0.11	96.7
30	Adenine	33.34	0.11	97.1
31	Quininic acid	33.67	5.71	89.6
32	Fructose	34.17	1.25	96.4
33	Galactose	34.28	21.88	77.2
34	Saccharide -unknown5	34.49	4.11	96.2
35	Mannitol	34.65	0.36	97.8
36	saccharide -unknown5	34.98	11.62	85.8
37	Gluconic acid	35.55	3.34	93.8
38	Ferulic acid	36.19	0.01	85.1
39	Myo-Inositol	36.50	8.37	96.6
40	Guanine	36.65	0.04	90.7
41	Caffeic acid	36.71	0.29	95.7
42	Tryptophan	37.60	0.04	83.1
43	Glyceryl-glycoside	38.69	0.58	94.5
44	Uridine	39.69	0.18	94.7
45	Sucrose	41.69	0.11	88.7
46	Cytidine	41.89	0.10	78.8
47	Trehalose	42.95	0.37	48.1
48	Chlorogenic acid	49.79	0.20	86.5
49	Cellobiose	50.97	0.09	83.2
50	Quercetin	51.17	0.06	88.3
51	Trisaccharide -unknown1	55.08	0.13	91.1
52	Trisaccharide -unknown2	55.86	0.02	68.8

## Data Availability

All data contained within the article.
